# Clinical and Radiological Evaluation of Surgical Treatment Outcomes in Disturbances of Scaphoid Bone Fusion in a Pediatric Population

**DOI:** 10.3390/jcm14113758

**Published:** 2025-05-27

**Authors:** Piotr Koschel, Leszek Kaczmarek, Jakub Woźniak, Piotr Czarnecki, Leszek Romanowski

**Affiliations:** 1 Department and Clinic of Traumatology, Orthopedics, and Hand Surgery, Poznan University of Medical Sciences, 61-545 Poznań, Poland; lkaczmarek@orsk.pl (L.K.); pczarnecki@orsk.pl (P.C.); lromanowski@orsk.pl (L.R.); 2 Lubuskie Centrum Ortopedii im. Dr. Lecha Wierusza w Świebodzinie, 66-200 Swiebodzin, Poland

**Keywords:** scaphoid, nonunion, non-vascularized, graft, adolescent

## Abstract

**Background/Objectives:** This study aimed to investigate the results of surgical intervention in cases of scaphoid nonunion and delayed healing among individuals aged 18 or younger, focusing on both clinical and radiological aspects, as well as identifying possible factors that may impact the effectiveness or failure of the treatment. **Methods**: A total of 20 boys with impaired scaphoid bone healing underwent surgical treatment, with the average duration between the suspected injury and the procedure being approximately 10.8 months. At the time of surgery, the patients had a mean age of 15 years, and the average follow-up period was 20 months. We assessed the type of surgery performed, along with range of motion and pain intensity, comparing preoperative findings with those recorded at the final evaluation. Based on lateral X-ray examination, CLA (capitolunate angle), SCA (scaphocapitate angle), and SLA (scapholunate angle) angles were measured before and after surgery and at the last follow-up. **Results**: The treatment results showed bone union in 18 out of 20 patients (90%), complete pain relief in 17 patients (85%), and a significant improvement in ROM. There was a statistically significant improvement in the SLA angle and an increase in wrist dorsal flexion. We also identified three factors that significantly influence better ROM after surgery, namely the values of the CLA and SLA angles, as well as the time from injury to surgery. **Conclusions**: Our findings suggest that surgical intervention contributes to improved joint mobility, pain reduction, and restoration of anatomical alignment in the wrist. While we identified factors associated with more favorable functional results, we did not find any that were linked to a higher likelihood of treatment failure.

## 1. Introduction

Scaphoid fractures are the most frequently occurring wrist fractures, representing 50–80% of all cases. In 5–10% of instances, healing is delayed, or a nonunion develops, which, if not properly managed, can lead to degenerative changes in the wrist over time [1]. Scaphoid fractures account for less than 1% of all fractures in individuals aged less than 18 years [2].

The diagnosis is based on X-ray examinations and further expanded by CT scans to estimate bone loss, assess vascularity, particularly of the proximal pole, and evaluate peri-scaphoid degenerative changes [3].

A scaphoid fracture most commonly affects the wrist, which can result in a bending of the scaphoid axis. Burgess [4], in his study, observed that axis disturbances significantly impact the range of dorsal flexion. Specifically, a 5% bend leads to an average loss of 24% of dorsal flexion. With a 15% bend, dorsal flexion is completely lost, with movement being possible only in the midcarpal joint. At a 30% bend, the trapezial bones are positioned on the proximal fragment of the scaphoid, resulting in a complete loss of wrist movement. This underscores the importance of restoring the proper scaphoid axis during surgery for nonunion, which is achieved by a graft. The graft may be vascularized, such as the 1,2-intercompartmental supraretinacular artery (1,2-ICSRA) pedicled graft, or by employing a free vascularized graft harvested from the medial femoral condyle (MFC). Another type of structural graft includes a corticocancellous autograft harvested from the distal radius or the iliac crest.

The scapholunate angle (SLA), which defines the anatomical relationship between the scaphoid and lunate bones, normally ranges from 30 to 60 degrees [5]. When this angle deviates from the normal range, it may indicate carpal instability, such as dorsal intercalated segment instability (DISI). In cases of displaced scaphoid fracture fragments, this angle may also be altered. The capitolunate angle (CLA) is the angle between the long axis of the capitate and the mid axis of the lunate on the sagittal imaging of the wrist. In a normal situation it should be <30° in the resting (neutral) position. The angle is increased in carpal instability such as with a DISI and volar intercalated segment instability (VISI). The scapholunate angle is used to differentiate between the two [6]. We also aimed to investigate whether scaphoid surgery affects the scaphocapitate angle (SCA).

In our study, the aim was to evaluate the surgical treatment of scaphoid nonunion in children. In the pediatric population, scaphoid nonunions are relatively rare, and the current literature on this topic remains limited. Moreover, children possess a greater biological healing potential due to better bone vascularity and regenerative capacity, which might distinguish the treatment from that of adults. Early scaphoid pathology in this age group is particularly important to study, as it may have long-term consequences for wrist function. We also assessed the impact of filling the defect with a cortical bone graft on restoring wrist congruity and range of movement.

## 2. Materials and Methods

In this study, we retrospectively evaluated the treatment outcomes of patients at the pediatric hand surgery unit in the traumatology, orthopedics, and hand surgery department of the Poznan University of Medical Sciences, Poland. As the data utilized were anonymous and part of routine clinical care, no specific consent was sought from patients. The study included patients treated in the department for scaphoid nonunion since 2016. We treated 20 boys with disturbances in the fusion of the scaphoid bone, with a mean time from the suspected injury to surgery of approximately 10.8 months. The average age at the time of surgery was 15 years. The mean observation time was 20 months. Patients with nonunion of the proximal pole were excluded, as well as those with incomplete data records. Seven patients who had the nonunion of the wrist of the scaphoid were disqualified from the study because they underwent procedures other than the use of a cortical bone graft from the iliac crest.

### 2.1. Surgical Technique

Under brachial plexus block, a volar approach to the scaphoid bone was performed. The nonunion site was debrided and resected until bleeding bone was visible. The size of the scaphoid defect was then measured by gently distracting the fragments using instruments while observing the deformity correction in the fluoroscopy. Subsequently, the hip was locally anaesthetized, and a bone graft of the appropriate size was harvested and placed into the nonunion gap. Stabilization was achieved using Kirschner wires (15 cases) or a Herbert screw (5 cases). The choice of stabilization method depended on the patient’s age—with Herbert screws more commonly used in older patients—as well as on the surgeon’s individual preference. The alignment correction and proper stabilization were verified under fluoroscopic X-ray guidance. The patient was then immobilized in a forearm–thumb spica plaster cast for at least six weeks.

### 2.2. Radiological Assessment

**Figure 1:**
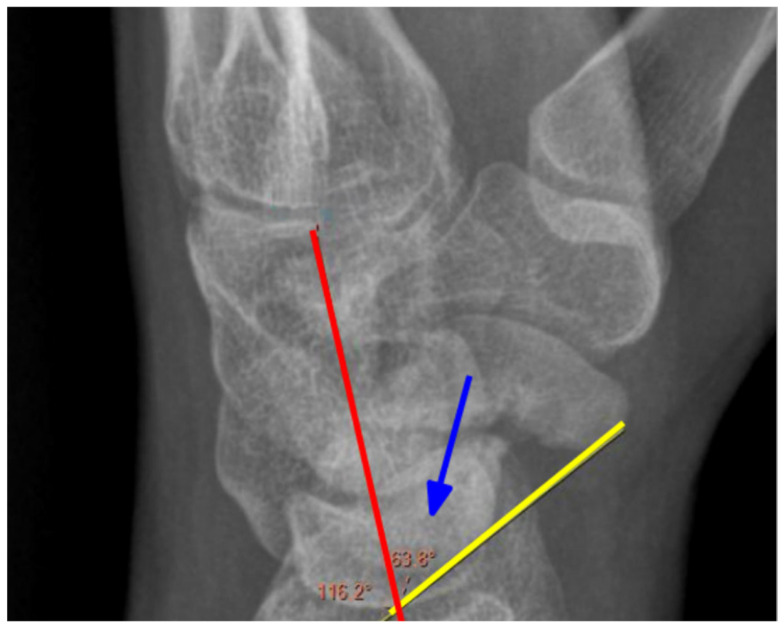
Measurement of the SLA angle. Blue arrow—angle, yellow line—the axis of the scaphoid, red line—the axis of the lunate. X-ray of the right hand, lateral view.

**Figure 2:**
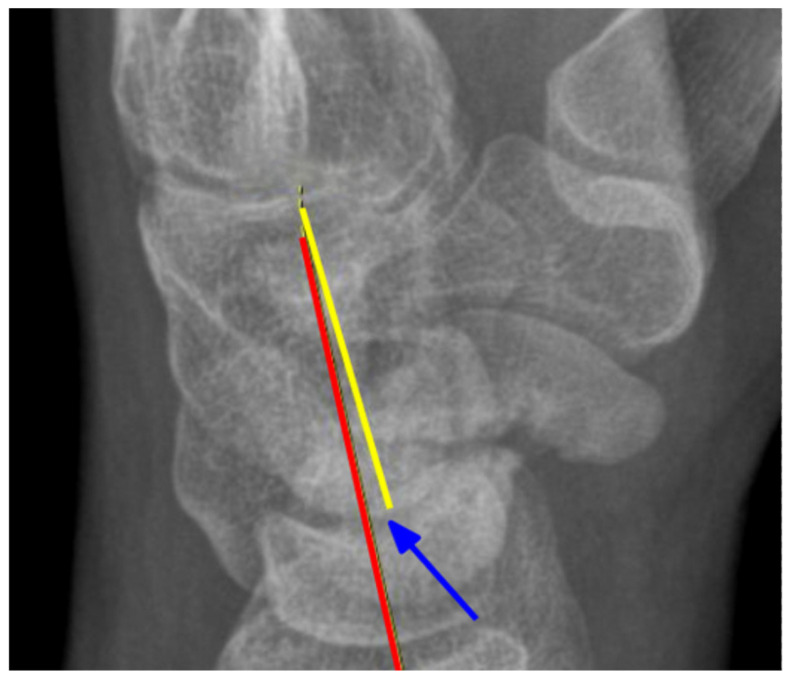
Measurement of the CLA angle. Blue arrow—angle, yellow line—the axis of the capitate, red line—the axis of the lunate. X-ray of the right hand, PA view.

**Figure 3:**
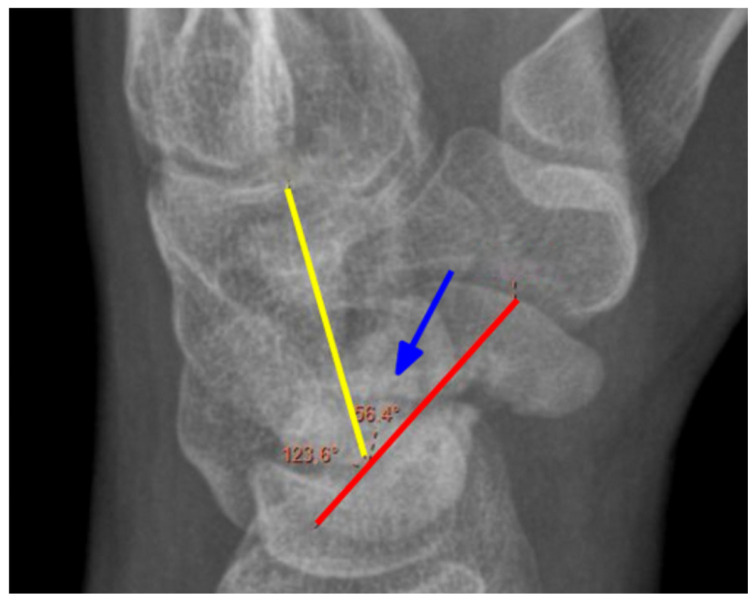
Measurement of the SCA angle. Blue arrow—angle, yellow line—the axis of the capitate, red line—the axis of the scaphoid. X-ray of the right hand, PA view.

Preoperatively, each patient underwent a CT scan to assess the nonunion and X-rays in three positions: postero-anterior (PA), lateral, and oblique. Postoperatively, surgical outcomes were evaluated the following day and during the final follow-up, where X-rays were taken in two projections: PA and lateral; in cases of nonunion or uncertainty, a CT scan was performed. Angles were measured retrospectively by two clinic physicians. The scapholunate angle (SLA) Figure 1, capitolunate angle (CLA) Figure 2, and scaphocapitate angle (SCA) Figure 3 were measured on lateral projections. The average of the two physicians’ results was used. Measurements were taken from imaging directly before and after surgery, as well as the latest available follow-up images. The SLA was measured between the axis of the lunate bone, determined by a perpendicular line to the line connecting its two prominences, and the line along the palmar surface of the scaphoid bone. The CLA was measured between the axis of the lunate bone and the axis of the capitate bone. The SCA was measured between the axis of the scaphoid and the capitate bones.

### 2.3. Statistical Analysis

Raw angle values were utilized for analysis. Angles were measured on the lateral Roentgen view. Values were described as within the normal range as follows: SLA 30–60, CLA < 30 [5], and for SCA, there is no normal range proposed. Consistency in the raw value measurements (preoperative, postoperative, and during the follow-up readings) obtained on the three separate occasions was assessed using the intra-class correlation coefficient (ICC). The reproducibility calculations were performed in R using the irr package. Each pair of angle measurements was averaged for subsequent analyses. Consistency of a single set of preoperative SLA, SCA, and CLA readings on 20 patients were compared to their postoperative and follow-up counterparts by computing the correlation of the raw measurements. From the measurements conducted by two authors, the averages for each measurement were calculated. The means were compared across repeated measurements with the ANOVA test. Due to the significance for the SLA and a result that suggested significance for the CLA, we performed the Tuckey test. We analyzed the variations in measurements across different time points. T1 represented the preoperative measurements, T2 the postoperative measurements, and T3 the final follow-up. We also examined the influence of factors such as the values of angles, the patient’s age at the time of injury, the time from injury to surgery on bone union, and the range of movement at the final follow-up. To assess these factors, we used linear regression models: one for the dependent variable “Union” and another for “ROM”. The dependent variable “ROM” was calculated as a percentage of the full range of wrist motion in all 20 patients, combining palmar flexion and dorsiflexion, 170 degrees total [7]. The independent variables included the values of individual angles before surgery, after surgery, and at the final follow-up visit, the patient’s age, and the time from injury to surgery. We also used the *t*-test to determine whether the changes in the range of dorsiflexion and palmar flexion motion before and after surgery were statistically significant in all patients. Statistical significance was established at the 5% level.

## 3. Results

**Figure 4:**
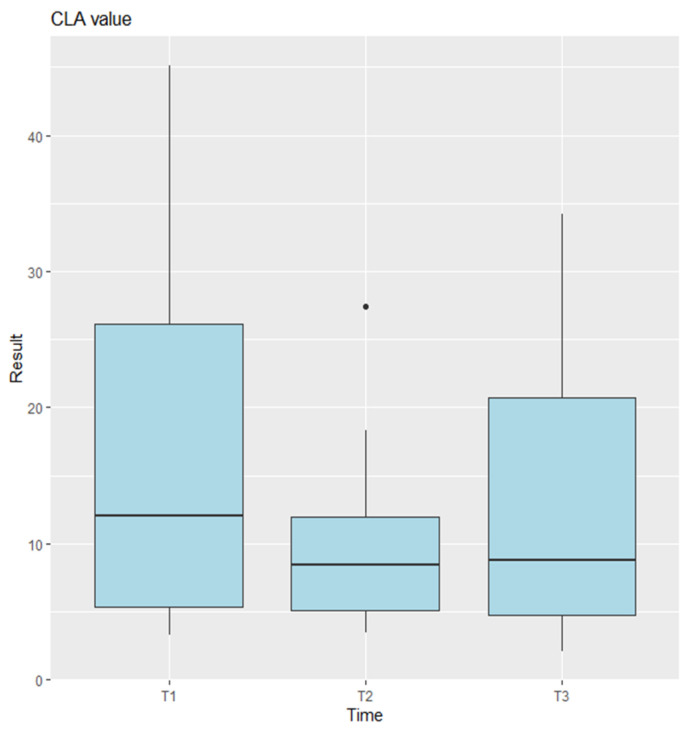
Anova results for CLA. T1 represents the preoperative measurements, T2 the postoperative measurements, and T3 the final follow-up. Points plotted outside the whiskers are considered outliers and represent data values that fall significantly below or above the typical range.

**Figure 5:**
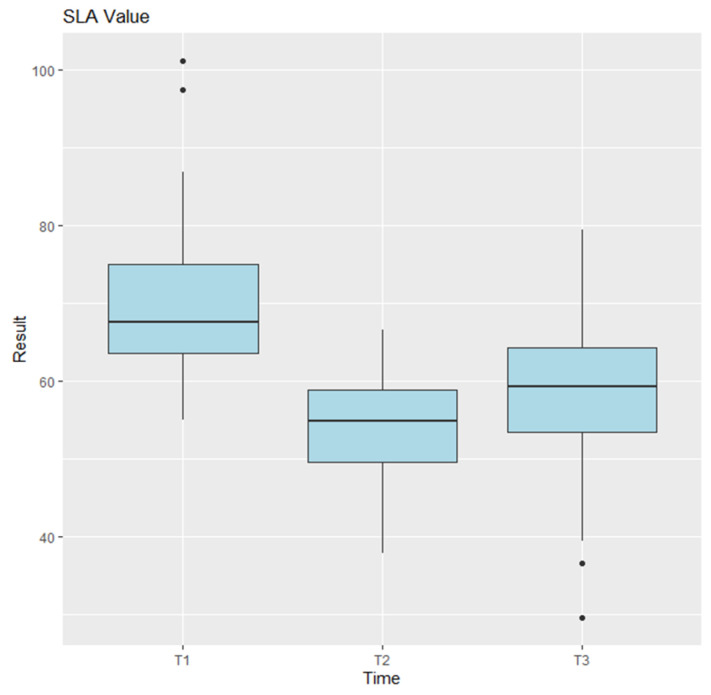
Anova results for SLA. T1 represents the preoperative measurements, T2 the postoperative measurements, and T3 the final follow-up. Points plotted outside the whiskers are considered outliers and represent data values that fall significantly below or above the typical range.

**Figure 6:**
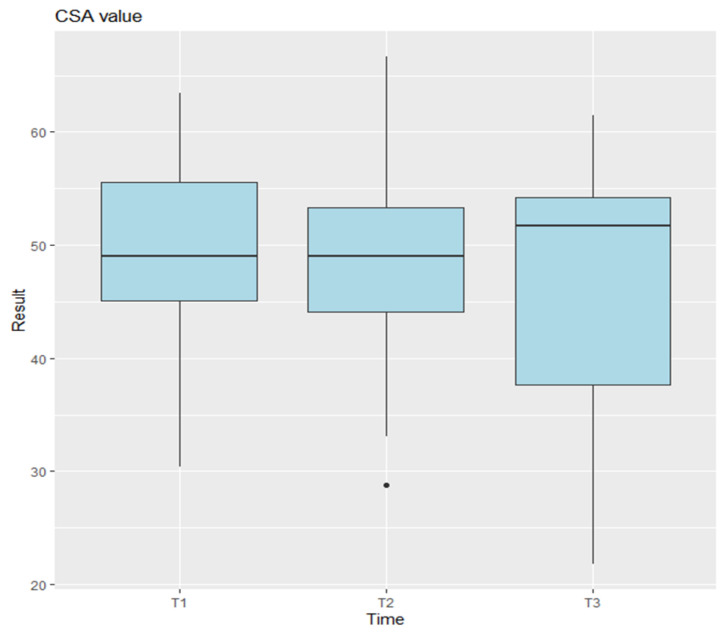
Anova results for CSA. T1 represents the preoperative measurements, T2 the postoperative measurements, and T3 the final follow-up. Points plotted outside the whiskers are considered outliers and represent data values that fall significantly below or above the typical range.

The CLA, SLA, and SCA were analyzed on preoperative, postoperative, and last follow-up imaging. Statistical analysis using ANOVA indicated no significant differences for the CLA measurements Figure 4 (*p* = 0.0745), and pairwise comparisons between time points were also not significant (T1–T2: *p* = 0.0705; T2–T3: *p* = 0.3884; T1–T3: *p* = 0.6219). In contrast, the SLA measurements showed highly significant differences (*p* = 9.47 × 10^−7^) Figure 5, with pairwise comparisons revealing significant differences between T1 and T2 (*p* = 0.0000096) and between T2 and T3 (*p* = 0.0004509), but no difference between T1 and T3 (*p* = 0.5129). For the CSA, ANOVA showed no significant differences (*p* = 0.541) Figure 6.

**Figure 7:**
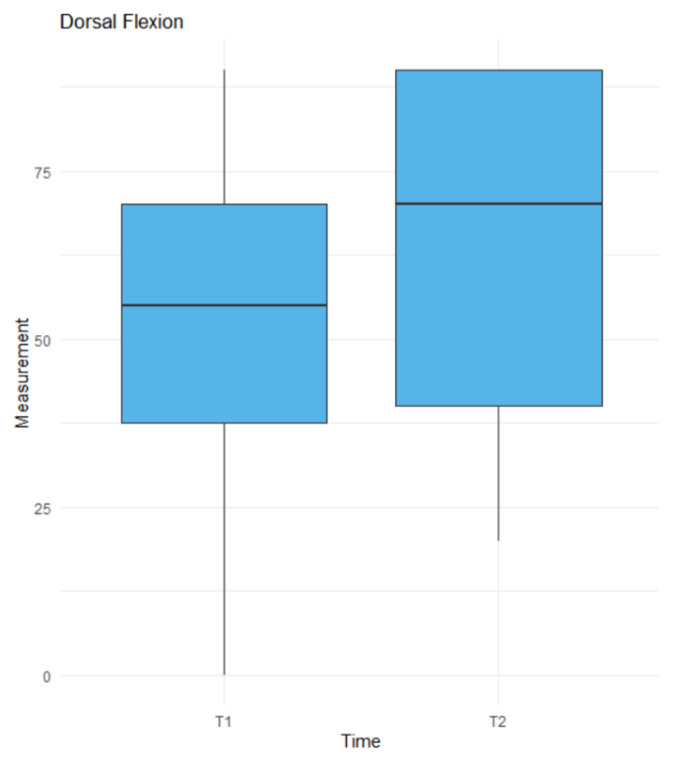
The results of the *t*-test for dorsal flexion in time. T1—before the surgery, T2—at the last follow up.

**Figure 8:**
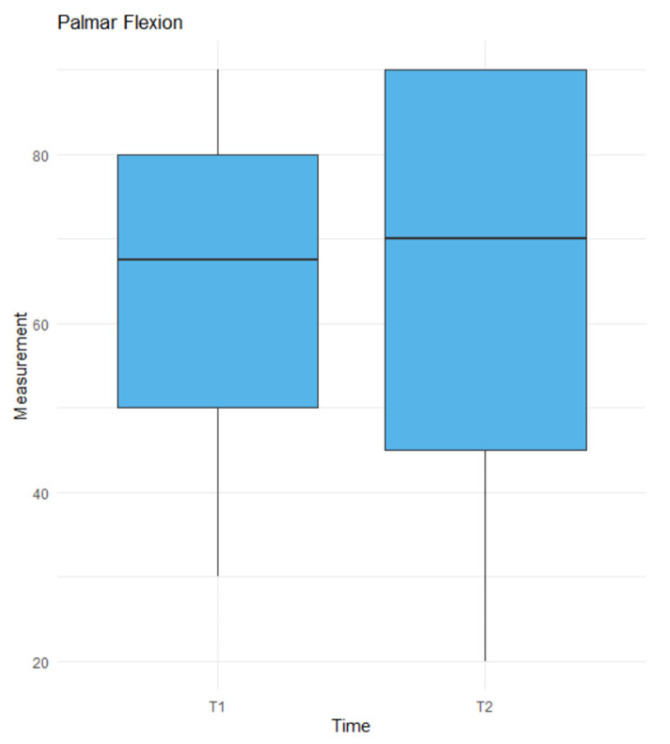
The results of the *t*-test for palmar flexion in time. T1—before the surgery, T2—at the last follow up.

The *t*-test revealed a statistically significant difference for dorsiflexion (*p* = 0.0424) Figure 7, whereas no significant difference was found for palmar flexion (*p* = 0.9671) Figure 8. Linear regression analysis identified CLA1 (*p* = 0.0296), SLA1 (*p* = 0.0112), and time (*p* = 0.0108) as significant factors influencing ROM Table 1. CLA1 had a positive effect, with a 1-unit increase was associated with a 2.47-unit increase in ROM. Conversely, SLA1 had a negative effect, with a 1-unit increase resulting in a 4.29-unit decrease in ROM. Time negatively impacted ROM, with each additional month from trauma to surgery reducing ROM by 2.46 units. Factors approaching significance included SCA2 (*p* = 0.0595), SLA2 (*p* = 0.0636), and SCA3 (*p* = 0.0723). Factors such as SCA1, CLA2, CLA3, SLA3, and patient age had no significant effect on the ROM. In the linear regression model for union Table 2, we did not find any factors with a *p*-value < 0.05 or approaching significance.

Clinical outcomes showed that 18 out of 20 patients (90%) achieved union, with an absence of pain in 17 patients (85%), with a mean follow-up period of 19.975 months (range: 3–51 months). Due to the retrospective nature of the study, it was not possible to assess all patients at the same time points. However, we believe that the follow-up period was sufficiently long to allow for the assessment of the maximal range of motion, evaluation of bone union or nonunion, and the detection of radiographic changes in the wrist. It is also one of the limitations of this study. Six patients (30%) underwent secondary procedures, including five who received autogenous bone marrow injections for delayed union and one who required removal of the headless bone screw due to nonunion. Among the patients who underwent revision surgery, bone union was achieved in four cases, and in two cases, we did not achieve union. No significant differences were observed between patients treated with Herbert screws and those treated with Kirschner wires. This analysis highlights the impact of radiological and temporal variables on patient outcomes and functional recovery.

## 4. Discussion

In our study, we aimed to present the outcomes of surgical treatment for scaphoid nonunion using non-vascularized bone grafts in adolescents.

Findings from this research indicate that surgery for a scaphoid nonunion does not affect the CLA and SCA angles. The SLA angle in our measurements significantly changed both after surgery and during the postoperative period. Despite this change, overall, we cannot conclude that our surgical treatment significantly affected the SLA angle, contrary to the findings of Israel et al. [2]. In another study [8], that also utilized iliac crest grafts, no significant changes were observed between postoperative measurements. However, the researchers found that carpal malalignment failed to correct in 32 out of 59 patients (54.2%) despite meticulous surgical technique and the placement of an appropriately sized wedge-shaped graft. Given our results and those of other studies, it would be advisable to expand the research and investigate which type of stabilization has the greatest impact on the change in the SLA angle. Moreover, the lack of significant improvement in the scapholunate angle (SLA) may indicate concomitant ligamentous injuries of the wrist, which could potentially lead to future instability. An increased SLA is indicative of dorsal intercalated segment instability (DISI). In cases where a postoperative improvement in the SLA is observed following bone grafting, but the angle subsequently returns to its preoperative value, this may suggest either graft resorption—supporting the use of vascularized bone grafts—or underlying scapholunate ligament instability.

Among the significant differences we reviewed in other studies, there is a slightly higher risk of avascular necrosis of the scaphoid after stabilization with a Herbert screw [9]. In our opinion, this also supports the argument for opting for Kirschner wire stabilization in underage patients. The aforementioned study also reports a statistically significant greater range of movement in patients stabilized with Kirschner wires. In a 2002 meta-analysis summarizing 36 studies [10], it was found that union was achieved in 94% of cases stabilized with Herbert screws and in 77% of cases stabilized with Kirschner wires. Our reported result regarding the union rate aligns with the findings of Munk and Larsen [11], who systematically reviewed 147 publications and observed a higher union rate in the K-wire group compared to the screw group. Specifically, the K-wire group demonstrated a higher estimated incidence of union (91% versus 88%, respectively), a difference that persisted when vascularized grafts were used (K-wire 94% versus screw 87%). A certain amount of axial interfragmentary displacement (IFD) has been shown to be acceptable and even beneficial in encouraging bone healing in long bone fractures [12], while transversal IFD has been clinically associated with nonunions. In the study conducted by Esquerro et al. [13], it was found that the minimum transversal IFD for fractures of the waist of the scaphoid treated via fixation with K-wires were computed by the models that simulated configurations with maximum gap between wires in the plane of fracture. In the model by Luria et al. [14], which examined the relationship between the type of scaphoid fracture, the positioning of the Herbert screw, and stability, the researchers found that greater force is required to generate scaphoid fracture displacement when screws are positioned centrally and perpendicular to the fracture than when they are positioned centrally, along the long axis of the scaphoid.

When comparing our findings to those of Israel [2], where they used vascularized grafts, the results are very similar (84.5% versus 90% in our population) regarding the likelihood of achieving union. The slightly different success rate may be attributed to a more diverse patient population, as well as the inclusion of individuals with proximal pole fractures of the scaphoid. It is worth emphasizing the relatively high number of patients (30%) who required a secondary surgical procedure; however, despite the need for reoperation, successful healing of the scaphoid nonunion was achieved in the majority of these cases (4/6). These secondary interventions were mostly bone marrow injections, we commonly use in the situations when the bone healing is delayed or union is not complete. No significant differences were observed between patients treated with Herbert screws and those treated with Kirschner wires. Therefore, based on this, we hypothesize that in children, the results are also similar when using either a vascularized or a non-vascularized bone graft. Due to the shorter surgical time, the absence of observed complications associated with harvesting a non-vascularized graft, and the lack of a clear advantage for vascularized grafts, we conclude that vascularized grafts have more limited indications. In the adult population, Hirsche et al. [15] achieved comparable results and suggests that vascularized bone grafts are recommended for patients with delayed treatment, compromised scaphoid vascularity, and revision surgery.

According to the statistical results of our study, surgery aimed to correct the scaphoid bone axis through grafting leads to a significant increase in dorsiflexion; however, it does not have an effect on palmar flexion. This finding aligns with Burgess’s results, which showed that a 5-degree deviation in the scaphoid axis results in a 24% loss of motion, and a 15-degree deviation leads to a complete loss of extension in the radiocarpal joint. Moreover, the statistical analysis revealed that a smaller initial SL angle and a larger CL angle are associated with greater postoperative range of movement, while a longer time from injury to surgery has a negative impact.

An aspect worth highlighting is the scaphocapitate (SCA) angle, a parameter that is scarcely reported in the current literature. Our findings suggest that the postoperative value of the SCA may have prognostic relevance and could potentially serve as an indicator of postoperative range of motion. Although this angle is not routinely assessed in clinical practice, its association with functional outcomes observed in our study indicates that it merits further investigation and consideration in future research and surgical planning.

Although previous studies confirm the greater reliability of wrist angle assessments using CT [16], once bone union is achieved, CT scans are only performed in uncertain cases. Therefore, we used available X-ray examinations for evaluation. This approach reduces the reliability of the results.

The limitations of this study include its small sample size and the absence of a comparative or randomized control group. The use of retrospective data introduces considerable limitations, including potential biases in data collection, incomplete documentation, and lack of standardized follow-up. We included patients with complete medical and radiological data to diminish the impact of these limitations on our study.

## 5. Conclusions

Our study demonstrates that surgical treatment has a positive impact on improving wrist range of motion, alleviating pain. Although surgical intervention leads to a significant initial improvement in the scapholunate angle, this correction tends to diminish substantially over the course of follow-up. While we identified several factors associated with better functional outcomes, no predictors of treatment failure were observed. Importantly, in summary, we can conclude that both vascularized and non-vascularized bone grafts yield comparable final results in pediatric patients. Therefore, the choice of grafting technique should be guided by the surgeon’s clinical judgment, experience and taking into account factors such as the presence or risk of avascular necrosis.

## Figures and Tables

**Table 1:** Results of linear regression analysis for individual factors affecting postoperative range of motion. The table includes estimates (β), standard errors, *t*-values, and *p*-values, indicating statistical significance. Abbreviation: ns—non significant, *—significant.

Variable	Estimate	Std. Error	*t*-Value	*p*-Value	Significance
(Intercept)	205.9359	69.2896	2.972	0.0178 *	* (*p* < 0.05)
CLA1	2.4792	0.9384	2.642	0.0296 *	* (*p* < 0.05)
SCA1	−0.6847	1.5438	−0.444	0.6691	ns
SLA1	−4.3850	1.3361	−3.282	0.0112 *	* (*p* < 0.05)
CLA2	−1.0177	0.9340	−1.090	0.3076	ns
SCA2	−1.3195	0.6013	−2.194	0.0595.	(*p* < 0.1)
SLA2	1.9726	0.9168	2.152	0.0636.	(*p* < 0.1)
CLA3	1.2620	1.1868	1.063	0.3187	ns
SCA3	3.3003	1.5947	2.070	0.0723.	(*p* < 0.1)
SLA3	0.8874	0.6099	1.455	0.1837	ns
Age	−3.5748	3.2291	−1.107	0.3004	ns
Time	−2.4581	0.7445	−3.302	0.0108 *	* (*p* < 0.05)

**Table 2:** Results of linear regression analysis for individual factors affecting union of the scaphoid. The table includes estimates (β), standard errors, *t*-values, and *p*-values, indicating statistical significance. Abbreviation: ns—non significant.

Variable	Estimate	Std. Error	*t-*Value	*p*-Value	Significance
(Intercept)	1.454671	1.537874	0.946	0.372	ns
CLA1	0.019640	0.020829	0.943	0.373	ns
SCA1	−0.018726	0.034264	−0.547	0.600	ns
SLA1	−0.029030	0.029654	−0.979	0.356	ns
CLA2	0.008902	0.020730	0.429	0.679	ns
SCA2	−0.010100	0.013346	−0.757	0.471	ns
SLA2	0.025260	0.020347	1.241	0.250	ns
CLA3	0.029833	0.026341	1.133	0.290	ns
SCA3	0.035614	0.035394	1.006	0.344	ns
SLA3	0.007634	0.013537	0.564	0.588	ns
Age	−0.078227	0.071669	−1.091	0.307	ns
Time	−0.015265	0.016523	−0.924	0.383	ns

## Data Availability

The original contributions presented in this study are included in the article. Further inquiries can be directed to the corresponding author.

## Abbreviations

The following abbreviations are used in this manuscript:

MDPI	Multidisciplinary Digital Publishing Institute
DOAJ	Directory of open access journals
CLA	Capitolunate angle
SLA	Scapholunate angle
SCA	Scaphocapitate angle
T1	Time: before surgery
T2	Time: just after surgery
T3	Time: at the last follow-up
